# Topology Optimisation for Compliant Hip Implant Design and Reduced Strain Shielding

**DOI:** 10.3390/ma14237184

**Published:** 2021-11-25

**Authors:** Nathanael Tan, Richard J. van Arkel

**Affiliations:** Department of Mechanical Engineering, Imperial College London, London SW7 2AZ, UK; natty135@gmail.com

**Keywords:** stress shielding, total hip replacement, femoral component, lattice, 3d printing, aseptic loosening, bone remodelling, internal structures, biomimicry

## Abstract

Stiff total hip arthroplasty implants can lead to strain shielding, bone loss and complex revision surgery. The aim of this study was to develop topology optimisation techniques for more compliant hip implant design. The Solid Isotropic Material with Penalisation (SIMP) method was adapted, and two hip stems were designed and additive manufactured: (1) a stem based on a stochastic porous structure, and (2) a selectively hollowed approach. Finite element analyses and experimental measurements were conducted to measure stem stiffness and predict the reduction in stress shielding. The selectively hollowed implant increased peri-implanted femur surface strains by up to 25 percentage points compared to a solid implant without compromising predicted strength. Despite the stark differences in design, the experimentally measured stiffness results were near identical for the two optimised stems, with 39% and 40% reductions in the equivalent stiffness for the porous and selectively hollowed implants, respectively, compared to the solid implant. The selectively hollowed implant’s internal structure had a striking resemblance to the trabecular bone structures found in the femur, hinting at intrinsic congruency between nature’s design process and topology optimisation. The developed topology optimisation process enables compliant hip implant design for more natural load transfer, reduced strain shielding and improved implant survivorship.

## 1. Introduction

Total hip arthroplasty (THA) is one of the most successful surgical procedures in modern medicine, providing a treatment option for debilitating diseases such as end-stage osteoarthritis and osteonecrosis of the femoral head. The majority of hip implants last for 25 years [1]. However, low failure rates still translate to many patients requiring revision surgery, as ~2 million procedures are performed worldwide each year [2]. For example, in the UK, 35,000 patients have required revision surgery [3]. These revision procedures cost more and have worse outcomes [4]. Hence, researchers and industry continually strive to advance technology to improve outcomes and survivorship.

Aseptic loosening is the most common reason for revision surgery [3]. One of the causes of aseptic loosening is strain shielding, which results from typical hip implant materials having moduli one to two orders of magnitude greater than the bone they replace/are implanted into [5,6,7]. Less load is transferred through the proximal femoral bone, resulting in a loss of the mechanical stimulus that drives bone formation. Over time, this leads to bone loss and increases the risk of loosening and/or periprosthetic fractures. It also increases the complexity of any revision surgeries as there is less bone available in which to implant a revision prosthesis.

The advent of commercial metal additive manufacturing provides the opportunity to manufacture hip stems with intricate cellular geometries, resulting in stems that are less stiff than their solid counterparts [7,8,9,10,11,12,13,14,15,16,17]. Most have adopted a lattice size optimisation algorithm, which is inherently limited by a minimum feature size. These designs are therefore subject to practical limiting factors, such as fatigue resistance and manufacturability of miniscule structures [18,19,20]. Indeed, researchers studying size-optimising lattices for femoral stems have commented that maintaining fatigue strength with small features is the most challenging criterion to fulfil when developing an optimal solution [9].

An alternative approach could be to borrow topology optimisation techniques from the field of structural optimisation [21,22] and directly use the results for hip implant design, without using lattice structures. As topology optimisation does not presume a priori material distribution, the algorithm has the liberty to leave specific locations void of material, whereas the corresponding size optimisation algorithm would be unable to remove unit elements from the lattice, merely reducing their sizes to a specified minimum. Such an approach has been demonstrated to reduced strain shielding in fracture plates [23], and has shown success in modelling the growth of internal bone structures when implemented with geometric constraints [24]. Thus, it holds great potential in producing implants that more closely match bone’s natural characteristics, reducing any strain shielding effect.

Topology optimisation is most used to minimise compliance (i.e., maximise stiffness) for a given volume fraction to provide mass reduction [22]. Thus, its application here might seem counterintuitive: Femoral stems are too stiff, hence there is need to increase compliance. The technique is useful, however, as imposing a volume fraction of less than one will always result in a concomitant increase in the minimum compliance when compared to a volume fraction equal to one. Thus, this approach will inherently produce more compliant stems despite maximizing the stiffness and strength at the set volume fraction. Indeed, the approach could enable a design tool for research across the risk/benefit spectrum by aiming for high/low global compliance objectives: A lower compliance target might enable more natural load transfer in the femur and reduce strain shielding but comes with risk that the design may be less robust against adverse loading scenarios (e.g., falls) and suboptimal implantations (e.g., stem undersizing [25,26]). Approaches to risk management vary between designers/manufacturers. Therefore, this research focuses on the development of the topology optimisation process rather than what the desired global compliance should be.

A factor that would aid translation would be preserving the outside shape of the hip stem. Implant manufacturers have spent decades optimising surgical instrumentation, interference fits, implant finishes and coatings. A design that maintains the outer shape of existing clinical implants could be advantageous as it could be implanted with minimal deviation from designs that have decades of clinical data supporting their use.

Therefore, this paper aimed to develop a topology optimisation process for designing the internal geometries of femoral hip stems for reduced strain shielding. Two proof-of-concept designs were manufactured and tested: (1) a porous implant, similar to those previously described but utilising a stochastic trabecular-like structure rather than cellular lattices, and (2) a novel selectively hollowed implant that maintains the outer shape of the implant such that only the stiffness of the stem is reduced, with no other design changes.

## 2. Materials and Methods

### 2.1. Topology Optimisation

#### 2.1.1. Solid Isotropic Material with Penalisation Method

**Figure 1:**
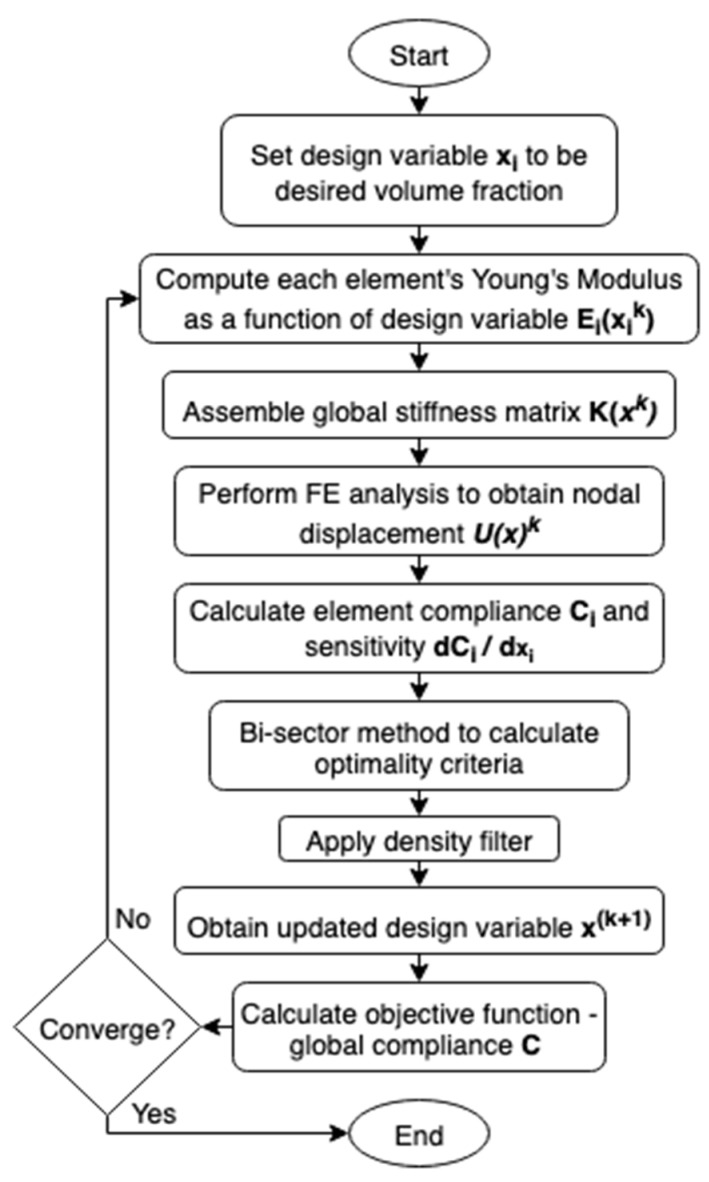
Overview of the Solid Isotropic Material with Penalisation optimisation method.

Femoral component topology was optimised via the Solid Isotropic Material with Penalisation (SIMP) method [21,22,27] (Figure 1). All elements were initially set to the desired volume fraction. Then, for each element in each iteration, the sensitivity of its compliance to change in volume fraction was calculated. The volume fraction of elements with sensitivity higher/lower than the optimality criteria was then increased/decreased, respectively, while ensuring the global volume fraction was maintained. The algorithm effectively removes material from the least sensitive element first, retaining maximum stiffness per unit of material deducted thus minimising the compliance of the model. 

A MATLAB function, TOP3D [27], was adopted and modified to develop the optimised hip implants. It performed SIMP topology optimisation on voxel models to find the design variables x of n total elements to minimise the structure’s compliance $c\left(\tilde{x}\right)$ while maintaining a material volume $v\left(\tilde{x}\right)$ less than or equal to a volume fraction $\bar{v}$. By default, the material distribution $\tilde{x}$ was the density-filtered design variables.


**Optimisation Problem Statement**
Find:

$x={\left[x_{1}\;x_{2}\;x_{3}\ldots \;x_{n}\right]}^{T}$

Minimize:

$c\left(\tilde{x}\right)=F^{T}U\left(\tilde{x}\right)=U{\left(\tilde{x}\right)}^{T}K\left(\tilde{x}\right)U\left(\tilde{x}\right)$

Subject to:

$v\left(\tilde{x}\right)=\;{\tilde{x}}^{T}v-\;\bar{v}\;\leq 0$



$x\in {\mathbb{R}}^{n}$



0≤x≤1



As the SIMP method changed each element’s stiffness based on design variables per iteration, the derivation of each element’s stiffness matrix was altered from conventional finite element methods:

(1) $$k^{0}=\int_{-1}^{+1}\int_{-1}^{+1}\int_{-1}^{+1}B^{T}C^{0}Bd\xi_{1}d\xi_{2}d\xi_{3}$$

Equation (1): Element stiffness matrix per unit Young’s modulus.

(2) $$E_{i}\left(x_{i}\right)=E_{min}+x_{i}^{p}\left(E_{0}-E_{min}\right)$$

Equation (2): Penalised element Young’s modulus.

(3) $$k_{i}\left(x_{i}\right)=E_{i}\left(x_{i}\right)k^{0}$$

Equation (3): Element’s Young’s modulus as a function of design variable.

Equation (1) was used to derive the element stiffness matrix per unit Young’s modulus using the deformation matrix for a unit cube B and constitutive matrix per-unit Young’s modulus $C^{0}$. Equation (2) was used to calculate each element’s Young’s modulus as a penalised interpolation between a defined minimum $E_{min}$ and material’s Young’s Modulus $E_{0}$ based on its design variable $x_{i}$ and the chosen penalisation factor p. Equation (3) was then used to compute each element’s stiffness matrix by multiplying the per-unit Young’s modulus stiffness by the element’s Young’s modulus. The sum of element stiffness matrices created the global stiffness matrix for that iteration. Standard finite element method steps were then taken to assemble the global force matrix F and calculate nodal displacements U.

During each iteration, each element’s design variable was adjusted incrementally based on the sensitivity of that element’s compliance with respect to its design variable. For elements with sensitivity greater than an optimality criterion, the design variable was increased, and those less than it decreased. The bisection method was used to determine the optimality criterion which yielded the minimum global compliance per iteration of the optimisation process.

The SIMP method often employs a density filter to mitigate numerical instabilities, such as the checkerboard problem, ensuring feasible solutions are obtained. Whereas the density filter ensures material continuity, it often results in greyscale solutions which require a non-uniform material Young’s Modulus to actualise. It has been proposed that filtering sensitivities, instead of densities, could provide a more black-and-white solution with minimal compromise on the optimality of solutions [28]. Being able to manufacture optimal solutions with minimal alterations and simplifications would both preserve the theoretical performance of the design and could enable patient-specific implants that could not only account for bone geometry [29], but also daily load conditions. Both the density and sensitivity filter were used to develop a greyscale and black-and-white solution of equivalent global stiffness. These solutions were developed into porous and selectively hollowed implants, respectively.

#### 2.1.2. Model Preparation

A Sawbones femur model (Model #3403, Sawbones) and a representative implant CAD model [30] were downloaded. A standard femoral neck resection was performed on the proximal femur, and the femur prepared for implantation by creating a cut out of the implant’s shape in the femur CAD model with Boolean subtract. The STL file was converted to voxel models in MATLAB using a ray intersection method [31]. As bone is an anisotropic material that comprises 2 distinctly different regions, a scanning function was also added to assign the outer cortical bone and inner trabecular bone with a Young’s modulus of 15 GPa and 0.8 GPa. The outermost surface of the implant was excluded from the optimisation to ensure continuity and contact between the implant and the femur.

#### 2.1.3. Loading Conditions

Previous work applying topology optimisation to the growth of a femur [24,32] highlighted the importance of considering the relative frequency of different load cases which simulate different daily activities. TOP3D was further modified to sequentially apply unique load cases with varying frequencies, simulating the peak hip joint reaction force during common activities of daily living [33] (Table 1). Muscles forces from the gluteal muscles and iliopsoas were added based on typical loads during gait [34,35,36] to demonstrate that muscle loading can be incorporated into the topology optimisation process. A load case of 1-1-1-2-3-4 (numbered in Table 1) was originally modelled, as prescribed by previous work [24]. However, this was later modified to a 1-2-1-3-1-4 sequence, as it was found that sequential gait loading led to premature convergence.

### 2.2. Implant Development

Implants were designed to achieve the same global compliance as a solid cortical bone implant. Whereas implants predominantly replace trabecular bone, the solid cortical implant reference was used as a proof of concept, exemplifying how the optimised implants can be tuned to a specific objective function. The reference objective function was obtained by calculating the compliance of a solid cortical bone implant subject to identical boundary conditions and one complete loading sequence. A parametric study was conducted to guide a trial-and-error process to produce optimal greyscale and black-and-white solutions that matched this global compliance objective function within 3%. 

#### 2.2.1. Porous Implant

**Figure 2:**
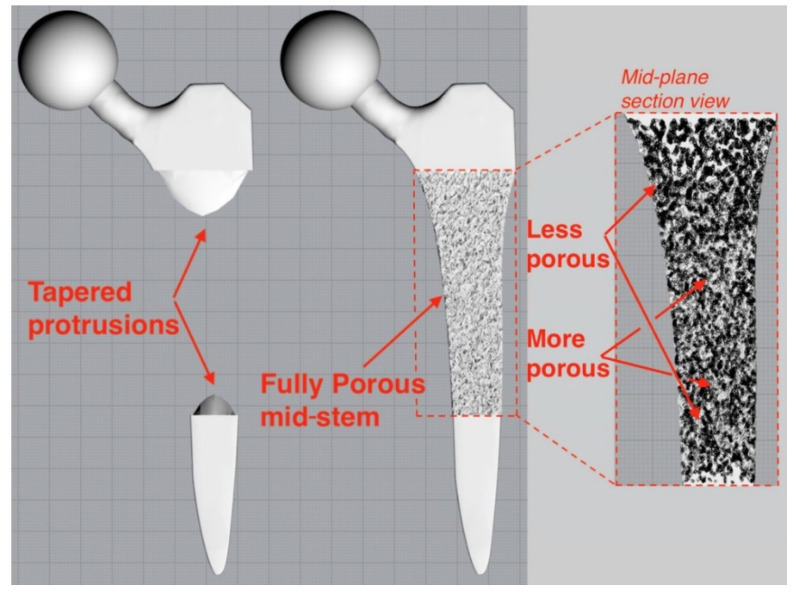
Tapered protrusions (left) enabled a gradual transition between the porous and solid regions of the porous implant (right).

The femoral stem was filled with a stochastic porous structure with infinitely thin stuts generated in Rhinoceros 3D (McNeel Europe, Barcelona, Spain). A charge field was then constructed and super-imposed over the strut array, where the magnitude of each point charge was determined by the design variable of the corresponding voxel from greyscale solution. Thicknesses were then prescribed to the struts depending on the charge field’s magnitude at each strut’s centre. The thicknesses were based on previous research which related laser parameters to strut thicknesses to mechanical properties to enable stiffness matched porous structures to be manufactured [19,37], leading to an implant design with 65% porosity and pore size range of 0.1–2 mm. The solid proximal and distal ends to the implant were then combined with the porous structure to finalise the implant (Figure 2). A tapering interface enabled a gradual transition between the porous and solid material regions.

#### 2.2.2. Selectively Hollowed Implant

**Figure 3:**
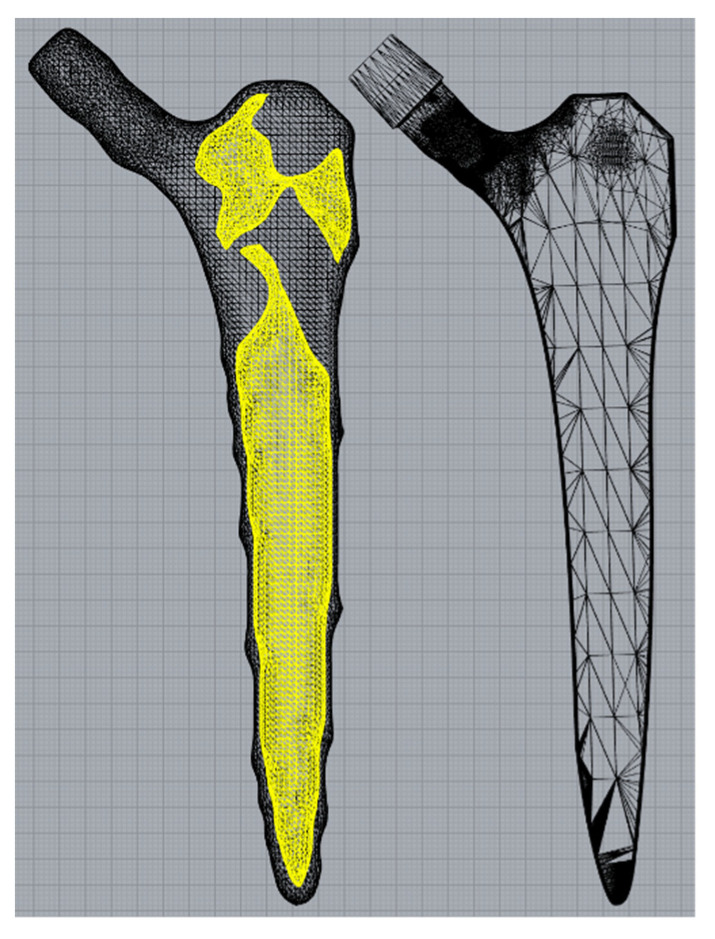
Internal geometry (yellow) separated from the rough external geometry (left) before being placed inside a standard implant (right).

The black-and-white solution was first converted to a STL file (Figure 3, left) using 3D Slicer [38] before the internal geometry was extracted and positioned within the standard implant model. Minor post processing was performed using Meshmixer (Autodesk Ltd, Birmingham, UK) to smooth kinks in the internal geometry and add distal drainage holes to enable non-fused powder removal post additive manufacture. The volume fraction of the of the hollowed stem was 40%.

### 2.3. Finite Element Analysis

Finite element analyses were performed in Fusion360 (Autodesk Ltd, Birmingham, UK). For all tests, the software’s automatic mesh generation and refinement feature was used with parabolic order elements. The initial element size was set to 5% of the model-based size, and the adaptive refinement control was set to high, with 10 iterations to achieve convergence within a tolerance of 5%. Von Mises stress was chosen as the convergence criteria (as opposed to displacement), as subsequent tests required stresses or strains to be investigated, and stresses tend to converge more slowly than displacements.

#### 2.3.1. Implant Strength

The strength of the novel selectively hollowed implant was compared to that of the solid stem through simulating BS ISO 7206-4-2010. The stem was angled 10°/9° in the coronal/sagittal planes, respectively, and a uniformly distributed vertical load of 2300 N was applied on the flat face of the femoral neck, producing a statically equivalent bending moment as a load applied through a spherical femoral head centre. First, stresses throughout the stem were observed visually to ensure no locations exceeded the yield stress of titanium. Second, maximum stress values were extracted and tabulated from two locations of interest: (1) at the femoral neck, as this is historically the weakest part of the hip implant; and (2) a transverse plane 10 mm proximal of the distal fixed face, away from the fixed boundary, at a location where the selectively hollowed implant had thin wall sections.

#### 2.3.2. Strain Shielding

The strain shielding stimulus was evaluated by quantifying peri-implanted strains following implantation of the solid and the novel selectively hollowed implants compared to the native femur. The native femur model was prepared by first transecting the complete sawbones femur model such that the distal tip of the femoral stem would be at least 10 mm from the fixed boundary condition applied to the distal femur. The femoral head was sliced at 45° in the superior-lateral direction, ensuring that the bending moment produced from forces applied on this surface would be equivalent to that produced by a point load in the centre of the femoral head. The trabecular bone and cortical bone regions of the sawbones model were again assigned a Young’s modulus of 0.8 GPa and 15 GPa, respectively, and the implants were inserted via Boolean subtract.

In total, 2300 N of load was applied to either the sliced femoral head (for the native femur) or the flat end of the implant (once implanted) in six loading directions (Table 2). The first load case represented the direction of gait loading most frequently imposed during optimisation, but with higher loading magnitude. Five other loading directions were chosen to investigate the correlation between reduction in strain shielding and loading direction, varying angles in each plane independently. These angles were based on the minimum, average and maximum angles of peak joint reaction force (JRF) [39] measured with instrumented hip implants during a range of daily activities [40], thus spanning the range of loads that may be expected during typical use. Importantly, these additional load cases meant that the implant’s performance was evaluated under load cases not used during the optimisation process.

Maximum principal strains were measured on the bone surface along the perimeter of the proximal-most plane for each of Gruen zones 4–7 [41]. Percentage strain was calculated by dividing peri-implant strain values with the native femoral strain values. It has been suggested that a reduction in strain of more than 50% (percentage strain < 50%) predicts bone resorption in that region [7]. By comparing percentage strain of the solid and selectively hollowed implants, improvements to strain shielding were quantified, and changes to trend along the length of the femur were observed.

### 2.4. Experimental Tests

Femoral heads were fitted to the stems in CAD, and the solid, selectively hollowed and porous designs were manufactured from 316L-0407 stainless steel powder in a powder bed fusion machine (AM250, Renishaw PLC, Gloucestershire, UK) in 50 μm layers. Different laser parameters were used for different regions of the stem: Porous structures were manufactured in line with previous research [19], while solid material was manufactured with standard laser parameters provided by the machine manufacturer and applied to the parts through their build preparation software (QuantAM, Renishaw PLC, UK).

**Figure 4:**
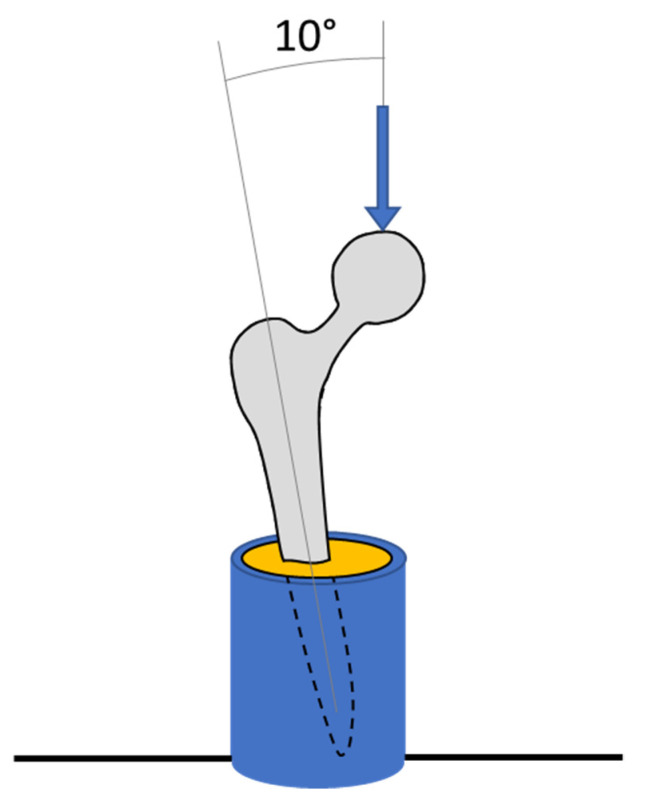
Diagram of experimental tests showing a hip implant (grey) secured in a metallic pot (blue) with PMMA (yellow).

For mechanical testing, the distal 40 mm of the implants was inserted into custom-made steel cylinders and held at 10° in the coronal plane using laboratory clamps. Poly methyl methyl acrylic (PMMA, Simplex, Kemdent) was poured into the cylinder, which, when cured, fixed the implant in this position (Figure 4). The steel cylinder had machined features that enabled it to attach to the base of a screw-driven materials testing machine equipped with a 5 kN load cell (model 5565, Instron, High Wycombe, UK). The fixture was positioned such that the femoral head was directly in the centre of a compression platen (accessory T1223-1021, Instron). Each sample was loaded up to 1500 N at a compression rate of 1 mm/min before being unloaded. Each sample was tested consecutively 3 times, and the average final deflection at 1500 N was calculated. The relative stiffness of each implant was calculated as the force applied per unit deflection of the femoral head.

## 3. Results

### 3.1. Stem Development

**Figure 5:**
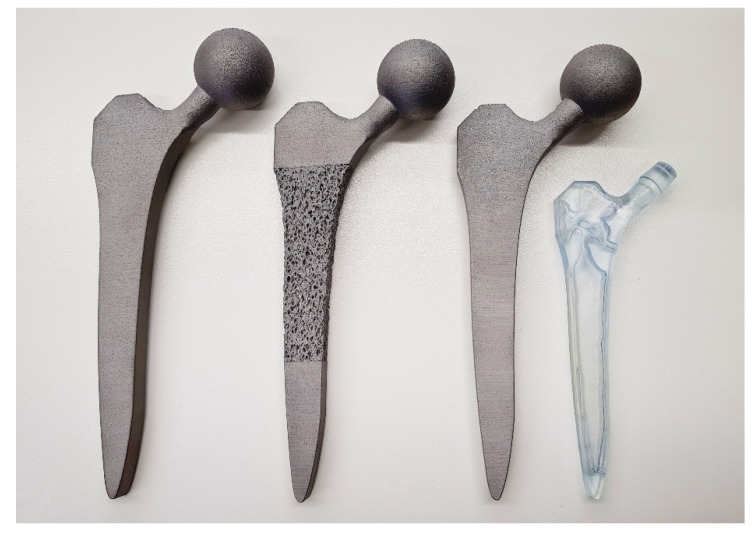
The successfully manufactured solid (left), porous (middle) and selectively hollowed (right) hip implants. The solid and selectively hollowed implants look identical. Thus, a clear resin plastic version of the selectively hollowed implant is also shown to indicate the internal structure of the selectively hollowed implant (right).

**Figure 6:**
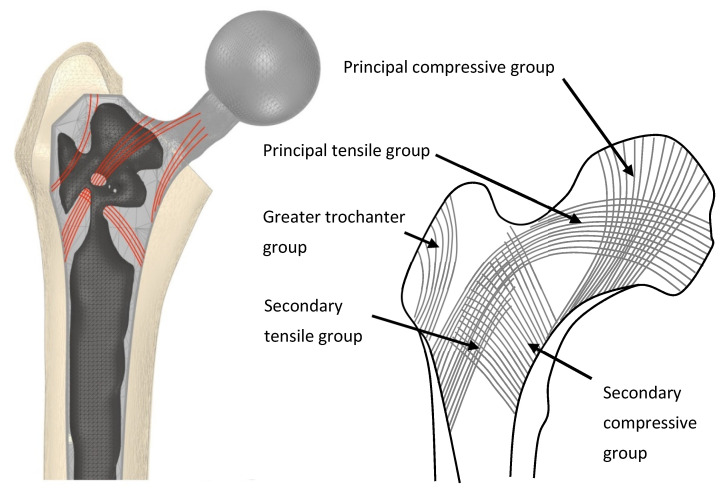
(Left) An example selectively hollowed stem implanted in a femur. Material remaining in the implant is indicative of the principal remodelling direction for trabecular bone in the femur (red lines). (Right) Representation of trabecular groups in the native femur.

Porous and selectively hollowed implants were successful designed and manufactured with the topology optimisation process (Figure 5). It was found that the 1-1-1-2-3-4 loading pattern led to premature convergence. Therefore, finite element analysis and experimental testing was only performed on the 1-2-1-3-1-4 loading pattern designs. Under this loading condition, the selectively hollowed implant had material distribution similar to the principal directions of the trabecular structure in the native femur (Figure 6).

### 3.2. Finite Element Analyses

#### 3.2.1. Strength

**Figure 7:**
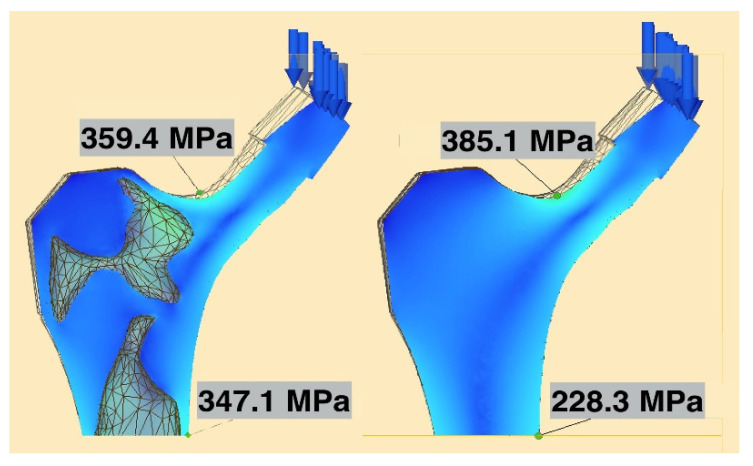
Maximum neck and stem stresses in the selectively hollowed (left) and solid (right) implants. The selectively hollowed implant had marginally lower neck stresses, but higher internal stem stresses compared to its solid equivalent. The image is cropped 10 mm from the distal fixed face.

**Figure 8:**
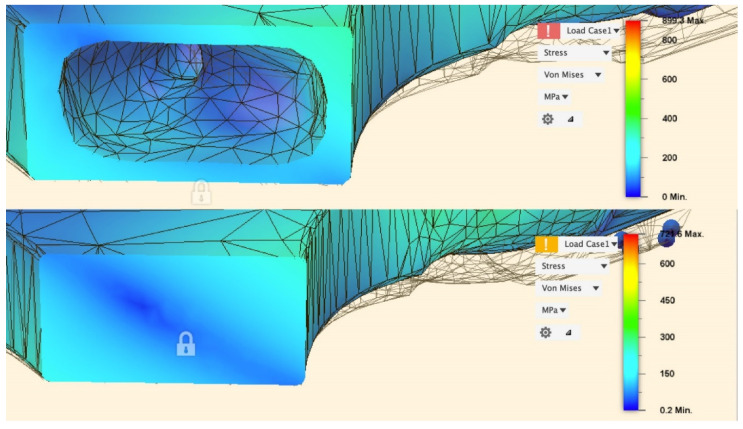
Stress distribution in the stem 10 mm proximal of the fixed face.

For both the solid and selectively hollowed implants, the maximum stress was located at the femoral neck (Figure 7), with the maximum stress marginally lower in the selectively hollowed implant. However, internal femoral stem stresses were approximately 50% greater in the selectively hollowed implant (Figure 7 and Figure 8).

#### 3.2.2. Strain Shielding

**Figure 9:**
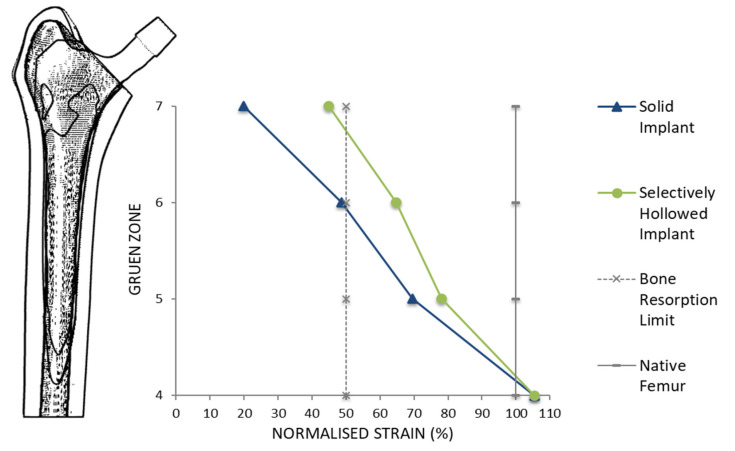
Peri-implant surface bone strain as a percentage of the strain measured for the native femur for different Gruen zones. A typical bone resorption limit of 50% was also indicated. The selectively hollowed implant strain shielded the femur less than the solid implant.

For the gait load case, both the solid and selectively hollowed implants were found to strain shield the femur, but the effect was less pronounced for the selectively hollowed implant (Table 3, Figure 9). The finite element analysis predicted that this exemplar selectively hollowed implant would lead to an 8-percentage-point (PP) reduction in strain shielding in Gruen zone 5, a 15 PP reduction in zone 6, and a 25 PP reduction in zone 7 compared to the solid implant.

**Figure 10:**
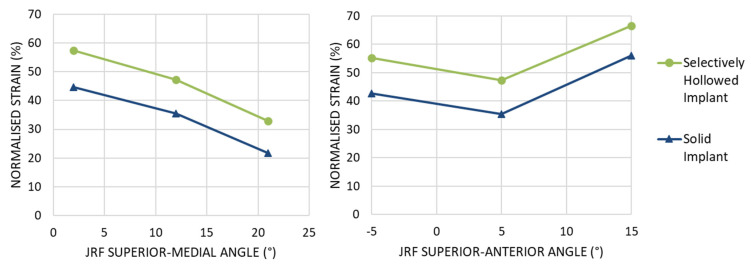
Peri-implant surface bone strain as a percentage of the strain measured for the native femur at Gruen zone 6 for different angles of joint reaction force (JRF). (Left) Variation in the coronal plane. (Right) Variation in the sagittal plane. For all load cases examined, the selectively hollowed implant resulted in less strain shielding than the solid reference implant.

The angle of the force affected the amount of strain shielding but not the percentage point difference between the implants. Increasing the angle of the implant in the coronal implant led to increased strain shielding for both implants, with a near constant percentage point difference between the two implant designs (Figure 10 left). Changing the angle of load in the sagittal plane resulted in a minimum at 5°, again with a near-constant percentage point difference between the two implant designs (Figure 10 right). For all load cases, the selectively hollowed implant reduced the strain shielding effect compared to the solid equivalent.

### 3.3. Experimental Stiffness Test

**Figure 11:**
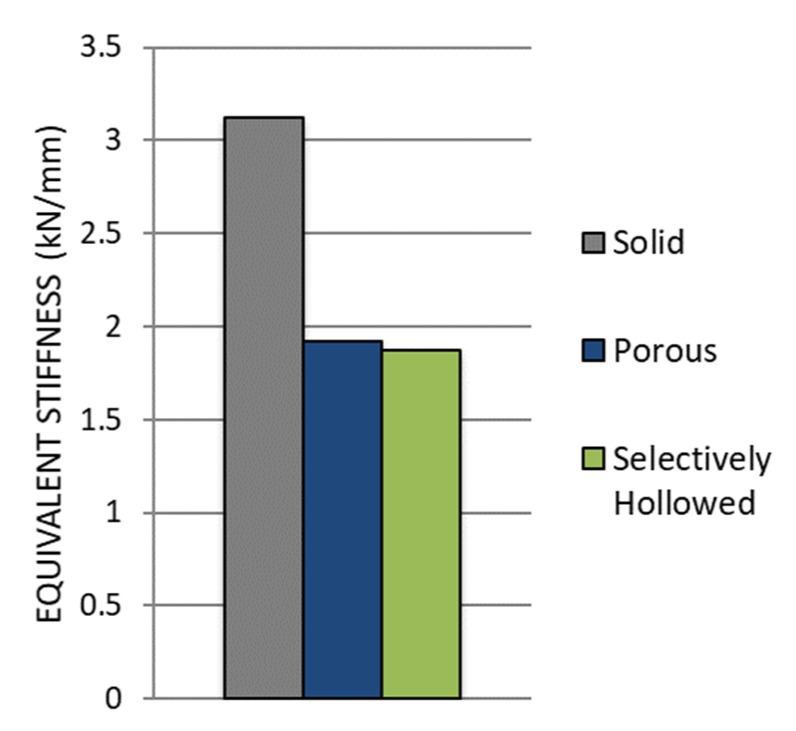
Comparison of experimental stiffness measurements for the solid reference, porous and selectively hollowed implant measured experimentally.

The porous and selectively hollowed implants were both more compliant than the solid reference implant: There was a decrease in the equivalent stiffness of 39% and 40%, respectively (Figure 11). There was minimal difference between the stiffnesses of the two compliant implant designs, with the porous implant being only 3% stiffer than the selectively hollowed implant.

The equivalent stiffness of the novel selectively hollowed implant was experimentally measured to be 1.9 kN/mm, which was 77% of the value predicted by the finite element analysis (2.4 kN/mm).

## 4. Discussion

This most important finding of this study is that topology optimisation can be used to design porous and selectively hollowed femoral hip stems with increased compliance for reduced strain shielding. The resulting stems had only a 3% difference in stiffness when additive manufactured despite the stark differences in their design. This demonstrates the broad scope for application of the topology optimisation approach presented and how different filters can be used to design different implant variations from an otherwise identical optimisation process. The developed process includes global optimisation for multiple consecutive load cases and enables either conservative or radical stem design through optimising to different global compliance objective functions.

Other research groups have found that lattice-based stem designs are effective for reducing strain shielding [7,8,9,10,11,12,13,14,15,16], with innovations including the inclusion of fatigue life constraints during the optimisation process [7,8,9]. The use of a trabecular-like stochastic porous structure in this study is potentially advantageous compared to more regular lattice designs as stochastic structures enable control of anisotropy [42], which may result in an implant that is more tolerant to unanticipated load cases. Other researchers have optimised the outer shape of hip implants [5,43,44,45,46,47,48], or optimised the modulus/density distribution within implants [49,50], with similar proposed benefits for preventing strain shielding. However, to the authors’ knowledge, this is the first paper to demonstrate that a strain shielding reduction can be gained by selectively hollowing the implant using topology optimisation with multiple load cases, and the first to additively manufacture and test a proof-of-concept device. The observed similarity between the selectively hollowed stem and trabecular structures (Figure 6) is likely explained by the inherent link between structural topology optimisation and the mechanisms that are believed to drive bone remodelling: The two concepts are mathematically matched [51]. Aside from its increased compliance and the need for a powder drainage hole, this novel selectively hollowed stem is indistinguishable from the solid counterpart (Figure 5), which may aid clinical translation, where each design change requires extensive testing and validation prior to clinical trial. For example, there is a complex interplay between broach design, technique and the stem to ensure suitable initial implant fixation [52,53]. Changing the outside shape of the stem would require the design of a new broach and re-validation to ensure that the desired fixation is achieved. Further, the selectively hollowed design inherently avoids transitions between porous and solid on the outer surface, which may lead to improved fatigue life compared to a porous stem by reducing the number of stress concentrations on the tensile face of the implant under bending loads.

The focus of this paper was to develop the multiple load case topology optimisation approach for hip implant design, rather than to produce a specific “best” design. Hence, simplifications were made to enable research to focus on the process rather than modelling complexity: (1) The bone was modelled with only a single property each for cortical/trabecular bone, whereas the bone was both inhomogeneous and anisotropic [54]. The process developed here could be applied to a calibrated CT scanned femoral bone model to capture regional variation in properties. (2) Loads based on only four daily activities were applied with loading from only two muscles. The activities simulated were based on previous works [24]. If a different set was chosen, or if activities had been included with greater temporal variation (in addition to just peak loading), then a different “optimal” stem would have resulted. Further, in vivo, there are 22 muscles spanning the hip joint, with load varying dynamically throughout activities [36]. The process developed is such that more physiological load cases can be added as desired, and future research could investigate which activities and phases of activity to include in the optimisation process. One might hypothesize that the inclusion of more physiological load cases would lead to a solution with greater similarity to the principal orientations and densities of trabeculae in the native femur. (3) The femoral stems were manufactured in 316L due to machine availability. Whereas this is a material used clinically, titanium alloy and cobalt-chrome alloy stems are more common. The topology optimisation process developed can inherently be used to find optimal stems in any material. (4) The FEA assumed that the implants and bone were fully bonded. This assumption represents an implant that has fully integrated into the body via bone in/on-growth [8] and has proven effective for predicting long-term bone remodelling [55,56]. Prior to this, implants are initially fixed via press-fit and friction [57], with bone growing into/onto the implant when the interfacial stress-strain state is appropriate and the relative micromotion between the implant and bone is around 110 μm [58]. Previous research into the effects of bone-prosthesis bonding has indicated that strain shielding is less during initial press-fit than when fully bonded [59]. As such, the fully bonded assumption made likely represents a worst-case scenario for strain shielding that is correlated with longer-term bone remodelling around the implant. (5) Machine compliance was not accounted for during the experimental stiffness tests. Further, stems were potted with PMMA (Figure 4) to fix them in the materials testing machine. PMMA has a modulus ~100 times less than steel leading to additional deformation under loading. Hence, the experimental result underestimated the stem stiffness, which helps explain the finding that the experimentally measured stiffness was 77% of the FEA predicted value. This does not affect the conclusions which focus on comparing the stems, rather than the absolute values achieved. Key factors, such as the orientation of the stem in the text fixtures, were controlled to ensure the validity of the relative comparisons made.

In conclusion, a topology optimisation process has been developed and applied to femoral hip stem design, resulting in two proof-of-concept designs that look radically different but achieve the same reduction in stiffness compared to a traditional solid implant. The approach can account for multiple load cases and enables design for different target global compliance. It provides an exciting new avenue for designing hip implants that enable more natural load transfer in the proximal femur for the reduced risk of strain shielding, bone loss and improved survivorship.

## Figures and Tables

**Table 1:** Magnitudes and directions of load cases 1–4.

Load Case	Action	Load (N)	Superior-Medial Angle (°)	Superior-Anterior Angle (°)
1	Walk	1925	17	11
2	Jog	3065	15	15
3	Sit down	1360	20	11
4	Stand up	1600	24	8

**Table 2:** Summary of the loading directions simulated strain shielding tests. The top row is equivalent to the peak gait loading angle used during the optimisation process. The other rows represent typical variations in the direction of the joint reaction force during activities of daily living.

Description	Superior-Medial Angle (°)	Superior-Anterior Angle (°)
Gait angles from optimisation	17	11
Min angle of JRF in coronal plane	2	5
Max angle of JRF in coronal plane	21	5
Average angle of JRF in both planes	12	5
Min angle of JRF in sagittal plane	12	−5
Max angle of JRF in sagittal plane	12	15

**Table 3:** Maximum surface bone strain and percentage of native bone strain for the selectively hollowed, solid implant and native femur for peak gait loading.

Model	Gruen Zone 4		Gruen Zone 5		Gruen Zone 6		Gruen Zone 7	
	Max Strain (με)	% of Native	Max Strain (με)	% of Native	Max Strain (με)	% of Native	Max Strain (με)	% of Native
Native femur	3040	100	2670	100	3010	100	2630	100
Solid Implant	3210	106	1860	70	1460	49	520	20
Selectively Hollowed Implant	3210	106	2080	78	1940	64	1180	45

## Nomenclature

B	Standard deformation matrix for a unit cube
$C^{0}$	Standard constitutive matrix per unit Young’s modulus
$c\left(\tilde{x}\right)$	Total compliance as a function of filtered design variable
$E_{0}$	Material Young’s Modulus
$E_{min}$	Non-zero minimum Element Young’s Modulus
$E_{i}$	Element Young’s Modulus
F	Global force matrix
$H_{ij}$	Weight factor ranging from 1 at the centre of element *i* to 0 at the centre of element j at a radius of R away from element *i*
$K\left(\tilde{x}\right)$	Global stiffness matrix as a function of filtered design variable
$k^{0}$	Element stiffness matrix per unit Yong’s modulus
$k_{i}$	*i*th Element stiffness
$N_{i}$	Neighbourhood of element *i*, defined as the volumetric space from centre of element *i* to centre of neighbouring element *j*
p	Penalisation factor
$U\left(\tilde{x}\right)$	Global displacement matrix as a function of filtered design variable
$v_{j}$	Unit volume per neighbour element *j*
$v\left(\tilde{x}\right)$	Total Volume Fraction as a function of filtered design variable
$\bar{v}$	Target volume fraction
$x_{i}$	*i*th Element design variable
$x_{j}$	Design variable of neighbour element *j*
$x_{i}^{p}$	Penalised ith element design variable
$\tilde{x}$	Filtered design variable
